# Spatiotemporal Dynamics of Plant–Soil–Enzyme Interactions in Intertidal Wetlands

**DOI:** 10.1002/ece3.73259

**Published:** 2026-03-14

**Authors:** Jiaxin Li, Wenwen Chen, Zulan Ou, Xingxing Yang, Lingling Li, Chunlin Li, Yansong Chen

**Affiliations:** ^1^ School of Resources and Environmental Engineering Anhui University Hefei China; ^2^ School of Biology and Food Engineering Hefei Normal University Hefei China

**Keywords:** exposure duration, intertidal wetland, plant community dynamics, Shengjin Lake

## Abstract

To investigate the spatiotemporal dynamics of plant communities in intertidal wetland during exposure periods, an in situ experiment with a two‐factor repeated measures experimental design was conducted in Shengjin Lake, China. We hypothesized that exposure duration would significantly affect plant community characteristics and associated soil properties. During the exposure period, 10 transects were established, with three quadrats evenly arranged at fixed intervals along each transect. Plant community characteristics, including species composition, coverage, and mean height, were measured repeatedly at early, mid, and late stages. Concurrently, soil samples were collected from the 0–20 cm layer to determine soil physicochemical properties and enzyme activities. Plant community composition differed significantly across both temporal (Global *R* = 0.728, *p* < 0.01) and spatial (Global *R* = 0.475, *p* < 0.01) gradients. With increasing exposure duration, ACP activity increased gradually, whereas TP showed an initial increase, followed by a decline. In contrast, EC, NH_4_^+^‐N, NO_3_^−^‐N, AK, TK, CAT, ALP, and UE decreased progressively. WC, pH, SOM, TN, AN, AP, and SC exhibited relatively minor variation. Redundancy analysis identified exposure duration as the dominant driver of plant community variation, followed by ALP, NO_3_^−^‐N, and pH. These results indicate that prolonged exposure enhances plant species diversity while modifying specific soil nutrient pools and enzyme activities. The coordinated temporal responses of soil nutrients and enzymes suggest an adaptive adjustment of the plant–soil–enzyme system to hydrological dynamics in intertidal wetlands.

## Graphical abstract

This study reveals that during the exposure period in the Shengjin Lake intertidal wetland, the temporal gradient serves as the primary driving force for changes in the plant–soil–enzyme system, followed by alkaline phosphatase activity, nitrate nitrogen, and pH. Consequently, plant communities exhibit significant differences along both temporal and spatial gradients, revealing the ecological mechanism by which hydrological changes shape plant community characteristics through the regulation of soil nutrients and enzyme activities.

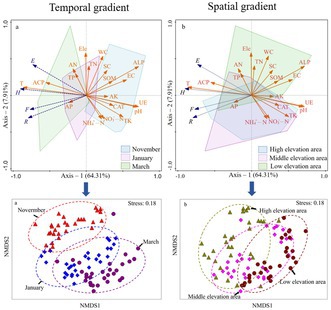

## 1 Introduction

Lake intertidal wetlands are vital ecosystems that play a crucial role in maintaining the ecological balance of lake environments. The systems exhibit distinct drying–rewetting dynamics driven by rhythmic hydrological fluctuations, which induce clear seasonal differentiation in plant communities across successive exposure stages (Huang et al. 2023). Such seasonal dynamics regulate ecological and evolutionary processes that shape species phenology. These processes determine species capacity to respond to variation in exposure duration (Hernández‐Carrasco et al. 2025). The exposed phase following water‐level recession represents a critical period for rapid ecosystem succession and key biogeochemical processes in intertidal wetlands. In the intertidal zone of the Three Gorges Reservoir, China, the life‐form composition of dominant plant species shifts from perennial to annual herbs as the exposure period extends from April to August (Zhang et al. 2020). Across both seasonal and interannual scales, exposure duration exerts pronounced effects on community diversity. In Poyang Lake, China, the biodiversity of Carex communities is significantly lower during years with prolonged exposure than during years with shorter exposure periods (Shen et al. 2020). Consistent with this pattern, other studies have reported declines in species richness and diversity with extended exposure periods (Antheunisse and Verhoeven 2008).

Plant community characteristics in intertidal wetlands also vary markedly across spatial scales, particularly along elevation gradients. In the Caohai Wetland, China, plant community richness index, Shannon–Wiener diversity, and abundance in low‐elevation zones are significantly lower than those in the middle‐elevation zones and high‐elevation zone (Qiu et al. 2023). A similar pattern has been observed in Lake Cerknica, where species richness increases with plot elevation (Gaberščik et al. 2018). Because water‐level gradients often closely correspond to elevation, these spatial trends are further supported by findings from Nyando floodplain wetland in Kenya, where plant species richness decreases with increasing water depth (Rongoei et al. 2014). Together, these studies demonstrate that plant community characteristics in intertidal wetlands exhibit distinct vary across temporal and spatial scales, from intra‐regional to inter‐regional levels, with significant differences in their dynamic patterns.

Wetland plants are core components of ecosystem function, and serve as sensitive indicators of environmental change (Maneas et al. 2019). Hydrological regimes can directly affect intertidal wetland plant communities (Lin et al. 2016; Ma et al. 2023; Liu et al. 2024) and indirectly shape species composition, spatial structure, and functional traits through changes in soil properties (Wu et al. 2021; Wan et al. 2024; Zhu et al. 2024). Significant associations exist between plant community characteristics and soil physicochemical properties in intertidal wetlands. In Delmarva Bay Wetlands, species richness and diversity decrease with increasing soil total nitrogen and total phosphorus concentrations (Russell and Beauchamp 2017). In the Yellow River intertidal zone, species diversity is primarily regulated by soil pH and soil temperature (Hong et al. 2021). In the Tonghui River wetland, the duration of inundation influences the distribution of dominant plant species through effects on soil water content, pH, and available potassium (Gao et al. 2024). Plant community characteristics are also affected by soil enzyme activities. Soil enzymes regulate biochemical processes by controlling microbial activity, for example by accelerating nitrogen and phosphorus mineralization through the decomposition of organic substrates (Heuck et al. 2015). These processes modify soil nutrient availability and physicochemical properties and thereby influence plant growth (Wu et al. 2023). Although previous studies have clarified spatial patterns or compared discrete exposure stages, the continuous co‐dynamics of plant communities and soil properties across the entire exposure period, and their mechanistic relationships over time, remain insufficiently understood.

Shengjin Lake, located in Anhui Province in the middle–lower Yangtze River basin, China, is a permanent freshwater lake hydrologically connected to the Yangtze River (Cheng et al. 2020), and provides critical habitat for waterbirds along the East Asian–Australasian migratory route (Wang et al. 2020). The lake exhibits strong seasonal variation in hydrological conditions (Wang et al. 2024). Water levels typically decline from November onward, allowing intertidal wetland vegetation to recover naturally. Levels rise again in April of the following year, resulting in widespread submergence and the disappearance of most wetland vegetation. Previous studies have shown that Shannon–Wiener diversity, species richness, and evenness during early exposure stages are primarily regulated by soil WC, TN, and carbon‐to‐phosphorus and nitrogen‐to‐phosphorus ratios (Zhang et al. 2021). However, the spatiotemporal dynamics of the integrated plant–soil–enzyme system along elevation gradients throughout the full exposure period remain unclear. Therefore, this study investigates the spatiotemporal dynamics and interaction mechanisms of the plant–soil–enzyme system across exposure stages and elevation gradients, focusing on: (1) plant community characteristics and their spatiotemporal dynamics; (2) temporal variation in soil physicochemical properties and enzyme activities; and (3) interactive relationships among plants, soil, and enzymes at different stages of the exposure period.

## 2 Materials and Methods

### 2.1 Study Area

**FIGURE 1:**
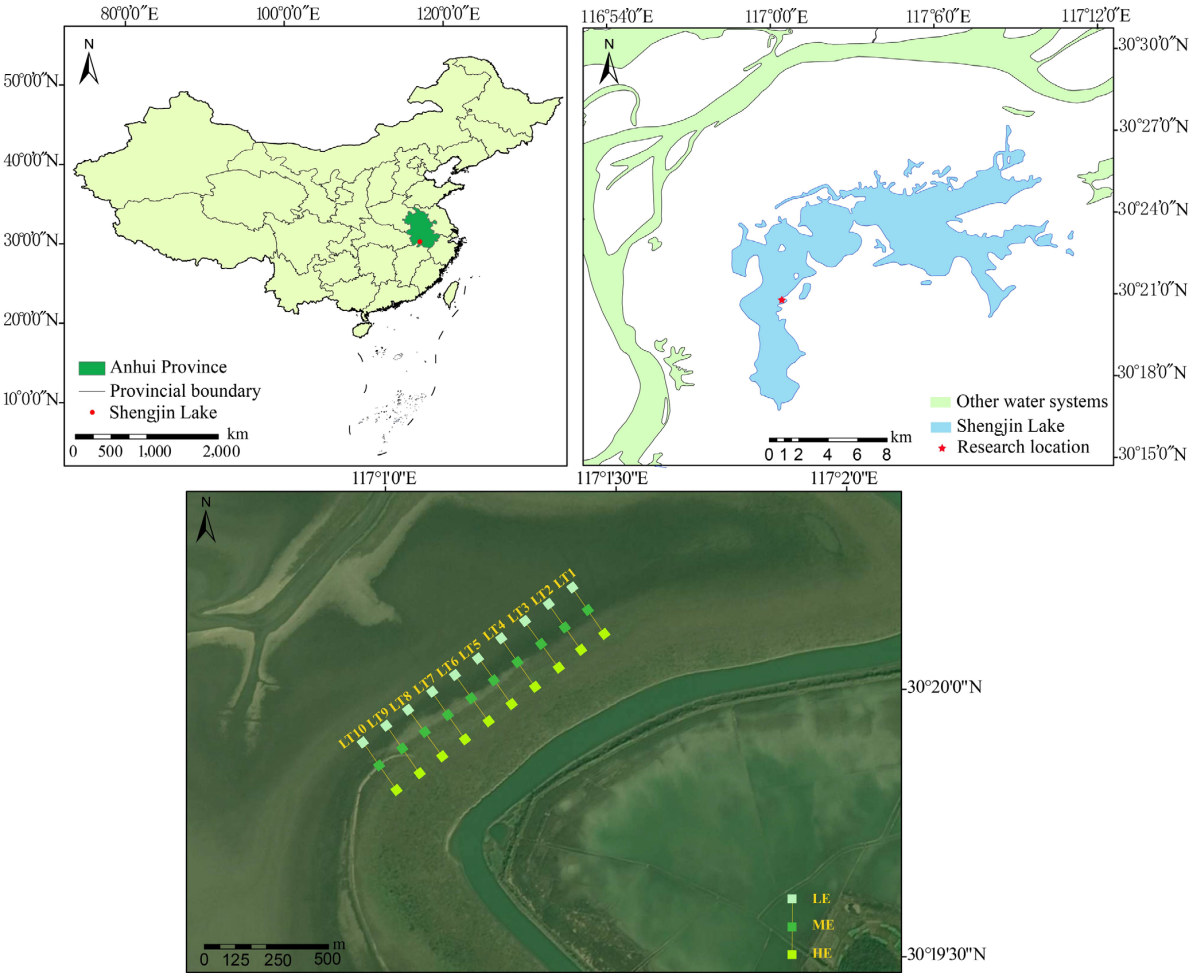
Study area and quadrat settings. LT1–LT10 represent transects 1–10. Squares indicate quadrats. LE, ME, and HE denote low‐, middle‐, and high‐elevation zones, respectively.

Shengjin Lake is located in a subtropical monsoon climate zone characterized by hot, humid summers and cold, dry winters. The mean annual frost‐free period is approximately 240 days. Mean annual precipitation is about 1600 mm, mean annual temperature is 16.1°C, and mean annual evaporation is approximately 757.5 mm. A representative section of the intertidal wetland of Shengjin Lake was selected as the study area (Figure 1). In this region, the intertidal exposure period generally extends from November to April of the following year. On the basis of plant community physiognomic characteristics, the exposure period was divided into three stages: early (T1), middle (T2), and late (T3). The corresponding mean temperatures were 16.71°C, 7.46°C, and 16.07°C, respectively. According to topographic features, the exposed intertidal zone was further classified into low elevation zone (LE), middle elevation zone (ME), and high elevation zone (HE), with mean elevations of 10.0, 10.5, and 11.0 m above sea level, respectively.

### 2.2 Plant Community Survey

Following the decline in lake water levels in autumn, the intertidal zone became exposed. Ten transects (LT1–LT10) were established parallel to one another along the elevation gradient from LE to ME to HE. Each transect was 200 m long, and adjacent transects were spaced 100 m apart. One herbaceous quadrat (1 × 1 m) was placed at each end and at the midpoint of every transect, yielding a total of 30 quadrats. Plant community surveys were conducted during the early, middle, and late stages of the exposure period. Species composition, coverage, and plant height were recorded in each quadrat, resulting in 90 sampled plots in total. Plant species were identified according to the *Flora Reipublicae Popularis Sinicae* (http://www.iplant.cn/frps). Coverage was estimated visually. The height of each species was measured in its natural state using a ruler with 1 mm precision.

For factorial analysis, duplicate soil samples were collected in parallel from a depth of 0–20 cm using a stainless‐steel auger and immediately placed in aluminum boxes (Φ 40 × 25 mm). All samples were transported to the laboratory in sealed containers. One set of samples was cleared of visible impurities and weighed to determine fresh mass. These samples were then oven‐dried at 105°C for 7 h to constant weight, defined as a mass difference of < 0.01 g between consecutive measurements, to determine soil WC. The second set of samples was air‐dried in a cool, well‐ventilated environment (25°C ± 3°C, relative humidity < 40%) for subsequent analyses.

### 2.3 Soil Variable Analysis

After removal of roots and gravel, soil samples were sieved through nylon meshes (60‐mesh and 100‐mesh) for the analysis of soil physicochemical properties and enzyme activities. Soil pH was measured using a pH meter with a soil‐to‐water ratio of 1:2.5. Soil electrical conductivity (EC) was determined using a DDS‐307 conductivity meter. A soil–water mixture at a ratio of 1:5 was shaken for 30 min and allowed to stand for an additional 30 min, after which it was filtered through qualitative filter paper. The conductivity meter was calibrated with a potassium chloride standard solution before measurement. Soil organic matter (SOM) was determined using the potassium dichromate oxidation–colorimetric method. TN was measured by the Kjeldahl method, and available nitrogen (AN) was determined using the alkali hydrolysis diffusion method. Ammonium nitrogen (NH_4_^+^‐N) was extracted with KCl and quantified colorimetrically at 625 nm. Nitrate nitrogen (NO_3_^−^‐N) was measured using the phenol disulfonic acid colorimetric method. TP was determined by the perchloric acid–sulfuric acid digestion method, and available phosphorus (AP) was extracted with 0.5 mol/L NaHCO_3_ and quantified. Total potassium (TK) was measured using the NaOH fusion–flame photometry method, and AK was extracted with NH_4_OAc and quantified by flame photometry. Soil enzyme activities, including catalase (CAT), alkaline phosphatase (ALP), acid phosphatase (ACP), sucrase (SC), and urease (UE), were measured using commercial assay kits produced by Nanjing Jiancheng Bioengineering Institute, China. Analytical procedures for soil physicochemical properties followed *Soil Agro‐chemical Analysis* (3rd ed.) (Bao 2000) and *Analytical Methods of Soil and Agricultural Chemistry* (Lu 2000).

### 2.4 Data Analysis

Species importance value (*IV*) was calculated to evaluate the relative importance of each species within quadrat communities (Jing et al. 2009; Tong et al. 2024). Plant community diversity was quantified using the Shannon–Wiener diversity index (*H*), Pielou evenness index (*E*), Margalef richness index (*F*), and richness index (*R*). The formulas were as follows:

Importance value (*IV*):

(1) $$\mathrm{IV}=\frac{\;\text{relative cover}+\;\text{relative height}}{2}$$

Shannon–Wiener diversity index (*H*):

(2) $$H=-\sum_{i-1}^{S}\;P_{i}\ln P_{i}$$

(3) $$P_{i}=\frac{n_{i}}{N}$$

Pielou evenness index (*E*):

(4) $$E=\frac{H}{\mathrm{lnS}}$$

Margalef richness index (*F*):

(5) $$F=\frac{\left(S-1\right)}{\mathrm{lnN}}$$

where $p_{i}$ is the importance value of the *i* species, $n_{i}$ is the number of individuals of the *i* species, *N* is *IV* of species, *S* is the number of species.

### 2.5 Statistical Analysis

SPSS 26.0 software was used to perform two‐way repeated‐measures analysis to assess soil variables across different exposure durations at the same elevation. One‐way ANOVA was applied to test differences in soil characteristics among exposure stages within the same elevation zone and among elevation zones within the same exposure stage. Statistical significance was set at *p* < 0.05. On the basis of quadrat survey data, the Shannon–Wiener diversity index (*H*), Pielou evenness index (*E*), Margalef richness index (*F*), richness index (*R*), and species importance values (*IV*) were calculated using Microsoft Excel 2017 (Microsoft Corporation, United States). One‐way ANOVA was employed to examine differences in plant community diversity indices among exposure stages within the same elevation zone and among elevation zones at the same exposure stage. Species importance values were log‐transformed as log(*x* + 1) using PRIMER 5 software (Plymouth Marine Laboratory, United Kingdom). A Bray–Curtis similarity matrix was constructed, and differences in plant community composition across elevations and exposure durations were tested using Analysis of Similarities (ANOSIM). Species contributing most to these differences were identified using SIMPER analysis. Because the gradient length from detrended correspondence analysis (DCA) was less than 3, redundancy analysis (RDA) was selected to identify key environmental factors. This analysis was performed using CANOCO 5 software (Microcomputer Power, Netherlands). Origin 2021 software (OriginLab Corporation, United States) was used to generate bar charts for ANOVA results.

## 3 Results

### 3.1 Spatiotemporal Dynamics of Soil Properties and Enzyme Activities During the Exposure Period

#### 3.1.1 Soil Physicochemical Properties

Exposure duration exerted significantly stronger effects on soil physicochemical properties than elevation (Table S1 in the Data S1). Two‐way repeated measures ANOVA showed that exposure duration had significant effects on EC (*F* = 55.30, df = 2, *p* < 0.05, $\eta^{2}=0.82$), WC (*F* = 8.20, df = 2, *p* < 0.05, $\eta^{2}=0.41$), pH (*F* = 26.28, df = 1.23, *p* < 0.05, $\eta^{2}=0.69$), AN (*F* = 19.01, df = 2, *p* < 0.05, $\eta^{2}=0.61$), NH_4_^+^‐N (*F* = 11.97, df = 2, *p* < 0.05, $\eta^{2}=0.50$), NO_3_^−^‐N (*F* = 9.48, df = 1.27, *p* < 0.05, $\eta^{2}=0.44$), TP (*F* = 16.73, df = 2, *p* < 0.05, $\eta^{2}=0.58$) and TK (*F* = 9.26, df = 2, *p* < 0.05, $\eta^{2}=0.44$). Elevation had no significant effects on any physicochemical indicators (*p* > 0.05). The interaction between elevation and exposure duration was significant only for EC (*F* = 3.11, df = 4, *p* < 0.05, $\eta^{2}=0.34$), AN (*F* = 2.94, df = 4, *p* < 0.05, $\eta^{2}=0.33$) and NO_3_^−^‐N (*F* = 5.30, df = 2.5, *p* < 0.05, $\eta^{2}=0.47$).

**FIGURE 2:**
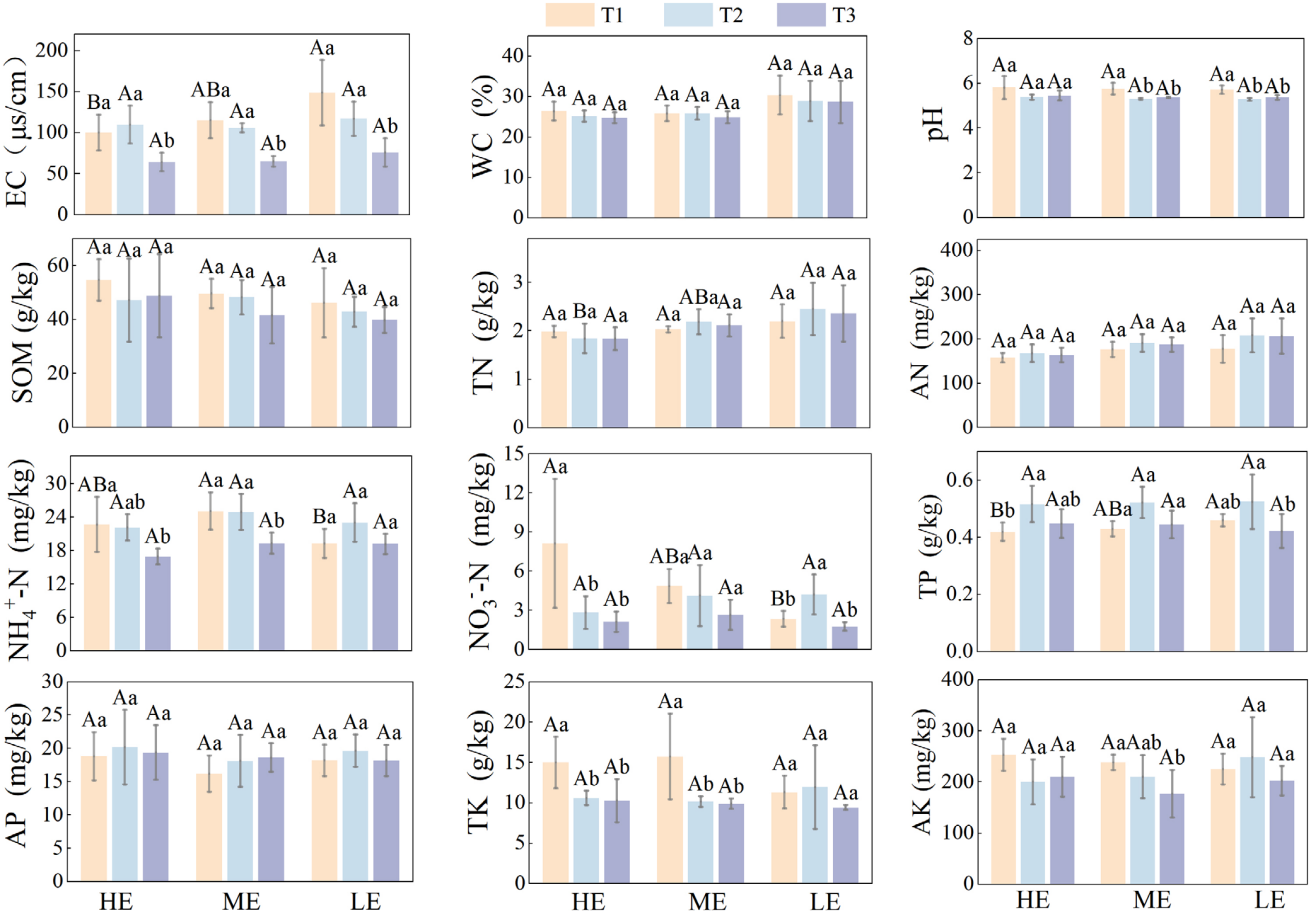
Effects of elevation and temporal variation on soil physicochemical properties during the exposure period in the Shengjin Lake intertidal wetland. Spatiotemporal dynamics are shown for three elevation zones, HE, ME, and LE, across early (T1), mid (T2), and late (T3) exposure stages. Soil properties include electrical conductivity (EC), water content (WC), pH, soil organic matter (SOM), total nitrogen (TN), available nitrogen (AN), ammonium nitrogen (NH_4_^+^‐N), nitrate nitrogen (NO_3_^−^‐N), total phosphorus (TP), available phosphorus (AP), total potassium (TK), and available potassium (AK). Statistical differences were evaluated using one‐way ANOVA with Tukey's post hoc tests. Capital letters indicate significant differences among elevations at the same exposure stage, and lowercase letters indicate significant differences among exposure stages within the same elevation (*p* < 0.05).

Temporal variation in soil physicochemical properties across elevation zones is shown in Figure 2. In HE, EC initially increased and then decreased, with values in T1 and T2 significantly higher than in T3 (*F* = 7.57, df = 12, *p* < 0.05, $\eta^{2}=0.56$). In both ME and LE, EC declined continuously, and values in T1 and T2 were also higher than those in T3 ME: (*F* = 19.16, df = 12, *p* < 0.05, $\eta^{2}=0.76$); LE: (*F* = 8.66, df = 12, *p* < 0.05, $\eta^{2}=0.59$). Soil pH decreased and then increased across all elevations. In ME and LE, pH in T2 was significantly lower than in T1 and T3 (ME: *F* = 12.98, df = 12, *p* < 0.05, $\eta^{2}=0.68$; LE: *F* = 15.81, df = 12, *p* < 0.05, $\eta^{2}=0.73$), whereas no significant were detected among elevations. NO_3_^−^‐N decreased continuously in HE, with T1 significantly higher than T2 and T3 (*F* = 6.06, df = 12, *p* < 0.05, $\eta^{2}=0.50$). In LE, NO_3_^−^‐N increased from T1 to T2 and then declined, with T2 significantly higher than T1 and T3 (*F* = 8.70, df = 12, *p* < 0.05, $\eta^{2}=0.59$). TP showed a unimodal pattern across all elevation zones, with T2 significantly higher than T1 and T3 in HE (*F* = 4.94, df = 12, *p* < 0.05, $\eta^{2}=0.45$) and ME (*F* = 6.03, df = 12, *p* < 0.05, $\eta^{2}=0.50$). Although SOM, AN, and AP showed numerical variation, the main effects of exposure duration, elevation, and their interaction were not statistically significant.

#### 3.1.2 Soil Enzyme Activities

Variation in exposure duration was the primary driver of soil enzyme activity dynamics, whereas the main effects of elevation and interaction effects were largely non‐significant (Table S2 in the Data S1). Two‐way repeated‐measures ANOVA indicated significant effects of exposure duration on ALP (*F* = 48.33, df = 2, *p <* 0.05, $\eta^{2}=0.80$), ACP (*F* = 20.87, df = 1.35, *p* < 0.05, $\eta^{2}=0.64$), and UE (*F* = 58.02, df = 1.24, *p* < 0.05, $\eta^{2}=0.83$), elevation only significantly affected SC (*F* = 5.89, df = 2, *p* < 0.05, $\eta^{2}=0.50$). No significant interactions between elevation and exposure duration were detected for any enzyme activities (*p* > 0.05).

**FIGURE 3:**
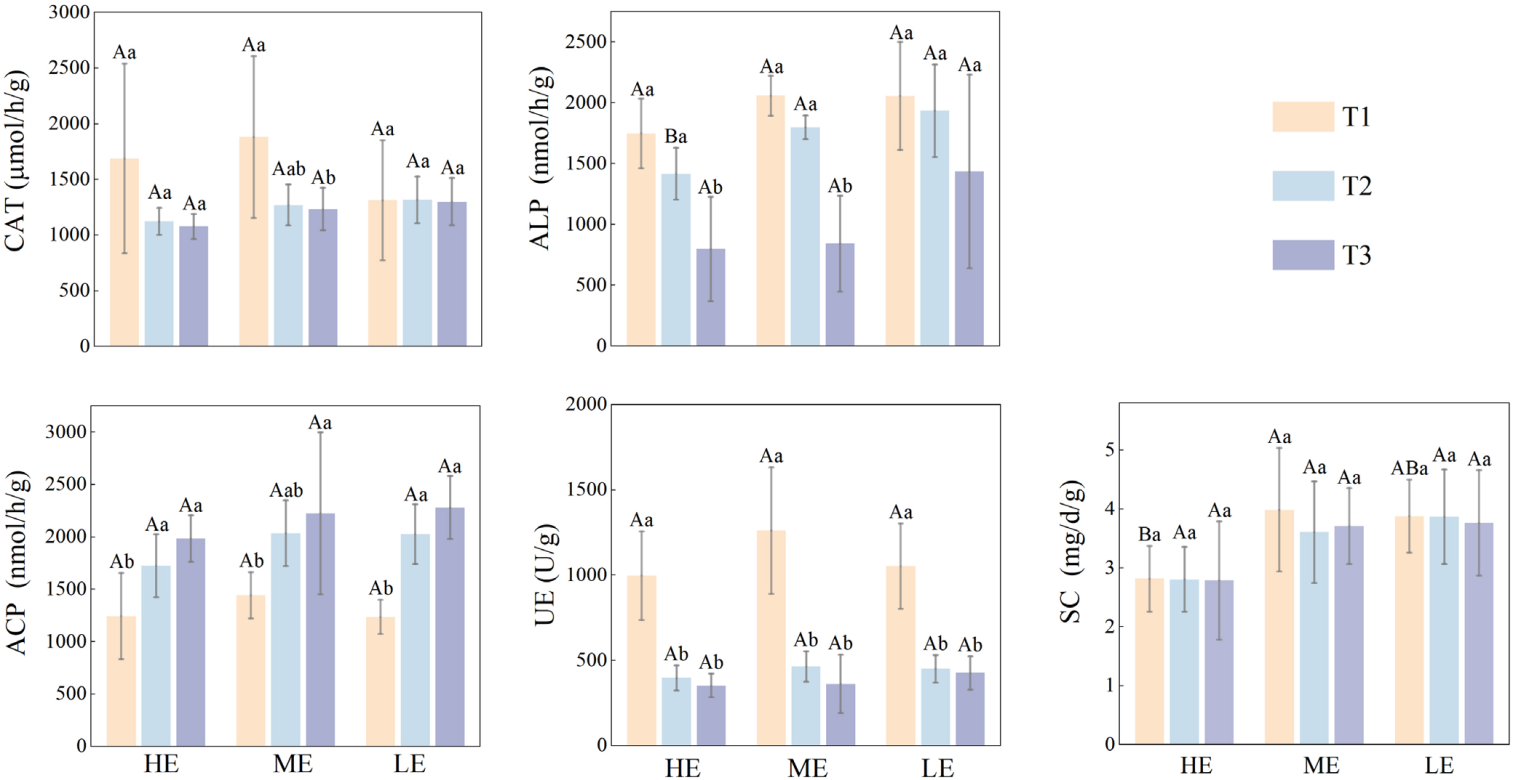
Effects of elevation and temporal variation on soil enzyme activities during the exposure period in the Shengjin Lake intertidal wetland. Spatiotemporal dynamics are shown for three elevation zones, HE, ME, and LE, across early (T1), mid (T2), and late (T3) exposure stages. Soil enzyme activities include catalase (CAT), alkaline phosphatase (ALP), acid phosphatase (ACP), urease (UE), and sucrase (SC). Statistical differences were evaluated using one‐way ANOVA with Tukey's post hoc tests. Capital letters indicate significant differences among elevations at the same exposure stage, and lowercase letters indicate significant differences among exposure stages within the same elevation (*p* < 0.05).

Temporal patterns soil enzyme activities across elevation zones are shown in Figure 3: ALP activity decreased continuously across all elevations, with T3 significantly lower than T1 and T2 in HE (*F* = 11.22, df = 12, *p* < 0.05, $\eta^{2}=0.65$) and ME (*F* = 32, df = 12, *p <* 0.05, $\eta^{2}=0.84$). ACP increased continuously across all elevations, with T1 significantly lower than T2 and T3 in HE (*F* = 6.86, df = 12, *p* < 0.05, $\eta^{2}=0.53$) and LE (*F* = 22.39, df = 12, *p <* 0.05, $\eta^{2}=0.79$). UE decreased continuously across all elevations, with T1 significantly higher than T2 and T3 in HE (*F* = 25.03, df = 12, *p <* 0.05, $\eta^{2}=0.81$), ME (*F* = 20.84, df = 12, *p <* 0.05, $\eta^{2}=0.78$) and LE (*F* = 24.10, df = 12, *p <* 0.05, $\eta^{2}=0.80$). SC activity differed significantly among elevations, with consistently higher values in ME and LE than in HE, whereas no significant effect of exposure duration was observed.

### 3.2 Spatiotemporal Succession of Plant Communities During the Exposure Period

#### 3.2.1 Plant Community Diversity

A total of 29 plant species belonging to 25 genera and 15 families were recorded in the intertidal wetland of Shengjin Lake (Table S3 in the Data S1). Asteraceae was the most represented family, comprising seven genera and seven species, followed by Polygonaceae with two genera and four species. Ranunculaceae, Lamiaceae, Rubiaceae, Fabaceae, and Boraginaceae each included two species, whereas the remaining families were represented by a single genus and species. *Carex thunbergii* occurred in all quadrats across the three surveys and exhibited the highest importance value, confirming its dominance in the study area.

Two‐way repeated measures ANOVA of plant community diversity indices showed that exposure duration had significant effects on the Margalef richness index (*F*) (*F* = 53.90, df = 2, *p* < 0.05, $\eta^{2}=0.67$), Shannon–Wiener diversity index (*H*) (*F* = 174.66, df = 2, *p* < 0.05, $\eta^{2}=0.87$), Pielou evenness index (*E*) (*F* = 83.63, df = 1.46, *p* < 0.001, $\eta^{2}=0.76$), and species richness (*R*) (*F* = 92.20, df = 2, *p* < 0.05, $\eta^{2}=0.77$) (Table S4 in Data S1). Elevation significantly affected *F* (*F* = 3.88, df = 2, *p* < 0.05, $\eta^{2}=0.22$) and *R* (*F* = 8.69, df = 2, *p* < 0.05, $\eta^{2}=0.39$). Significant interactions between elevation and exposure duration were detected for *H* (*F* = 3.54, df = 4, *p* < 0.05, $\eta^{2}=0.218$) and *E* (*F* = 3.18, df = 2.92, *p* < 0.05, $\eta^{2}=0.19$).

**FIGURE 4:**
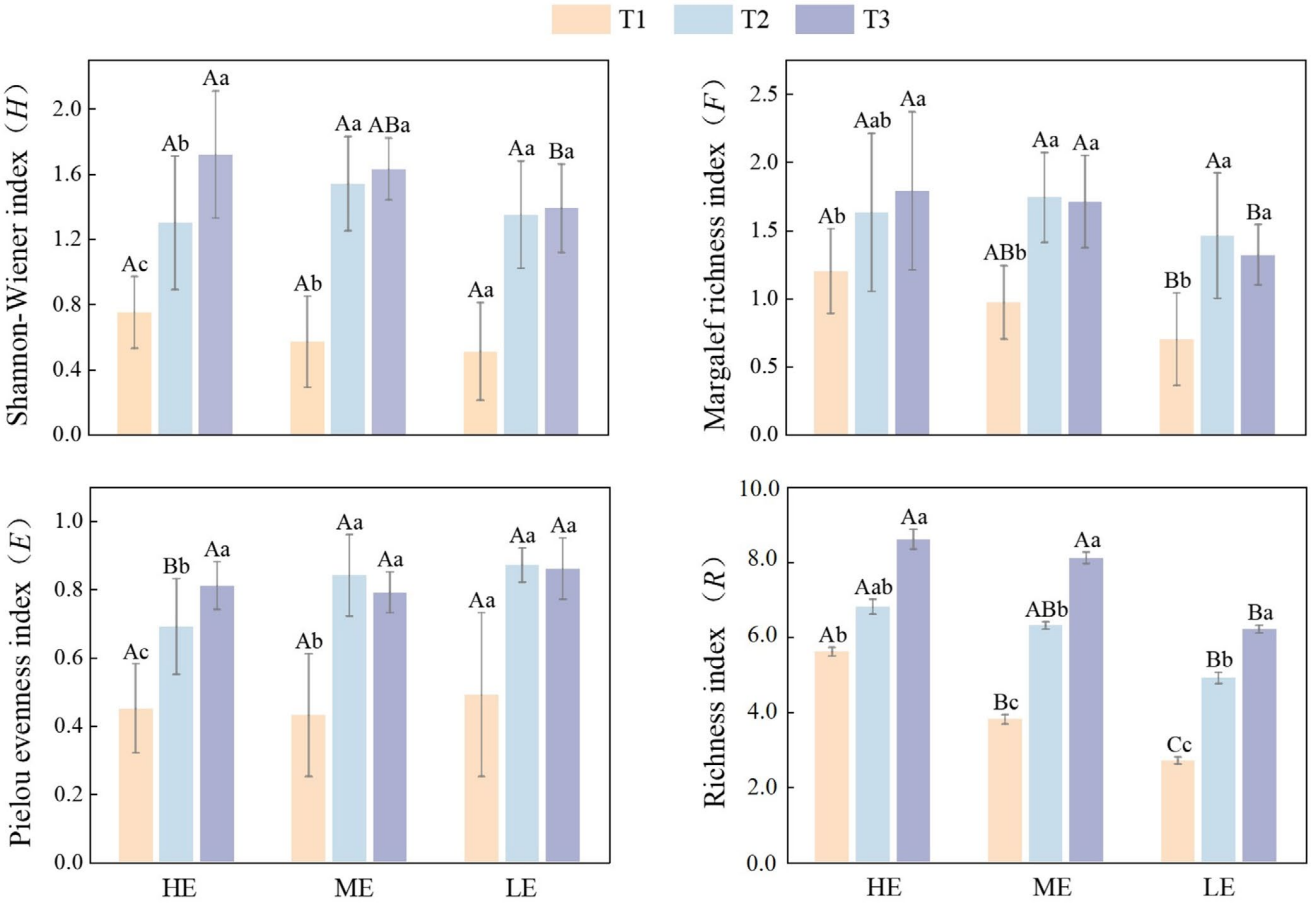
Effects of elevation and temporal variation on plant community diversity indices and species richness during the exposure period in the Shengjin Lake intertidal wetland. Variations in the Shannon–Wiener index (*H*), Margalef richness index (*F*), Pielou evenness index (*E*), and richness index (*R*) are shown for HE, ME, and LE elevations across early (T1), mid (T2), and late (T3) exposure stages. Statistical differences were assessed using one‐way ANOVA with Tukey's post hoc tests. Capital letters indicate significant differences among elevations at the same exposure stage, and lowercase letters indicate significant differences among exposure stages within the same elevation (*p* < 0.05).

Within each elevation zone, *H* and *R* increased significantly with exposure duration. *F* and *E* exhibited similar temporal trends. Overall, the highest values of *H* (1.72 ± 0.39) and *F* (1.79 ± 0.58) were observed in the HE in March, whereas the lowest values (0.51 ± 0.30 and 0.70 ± 0.34, respectively) occurred in the LE in November. The highest value of *R* (8.6 species) was recorded in HE in March, whereas the lowest value (27 species) occurred in LE in November. The highest value of *E* (0.87 ± 0.05) was observed in LE in January, and the lowest value (0.43 ± 0.18) occurred in the ME in November (Figure 4).

#### 3.2.2 Plant Community Similarity

**FIGURE 5:**
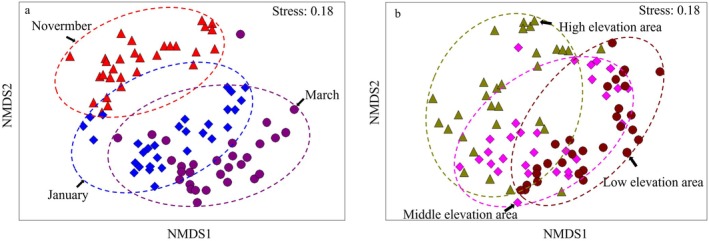
Nonmetric multidimensional scaling analysis of plant communities in the Shengjin Lake intertidal wetland. NMDS illustrates plant community dissimilarities across exposure stages, early (T1), mid (T2), and late (T3), and across elevation zones, HE, ME, and LE. In the temporal analysis (a), T1, T2, and T3 communities are indicated by △, ◇, and ○, respectively. In the spatial analysis (b), HE, ME, and LE communities are indicated by △, ◇, and ○, respectively. A stress value of 0.18 indicates an acceptable two‐dimensional representation of community relationships.

Two‐way crossed ANOSIM revealed significant effects of exposure duration (Global *R* = 0.728, *p* < 0.01) and elevation (Global *R* = 0.475, *p* < 0.01) on plant community composition. Non‐metric multidimensional scaling (NMDS) further demonstrated clear separation of communities by exposure stage and elevation (stress = 0.18) (Figure 5). Along the temporal gradient, the greatest dissimilarity was observed between T1 (November 2023) and T3 (March 2024) (Global *R* = 0.889, *p* < 0.01), whereas the difference between T2 (January 2024) and T3 was smaller (Global *R* = 0.576, *p* < 0.01). Along the spatial gradient, the highest dissimilarity occurred between LE and HE (Global *R* = 0.695, *p* < 0.01), while dissimilarity between ME and LE was lower (Global *R* = 0.358, *p* < 0.01).

The SIMPER analysis quantified the contributions of species to dissimilarities among assemblage groups derived from the Bray–Curtis similarity matrix. Nine dominant species together accounted for more than 80% of the total dissimilarity among the six groups (Table 1). *C. thunbergii* contributed more than 40% to relative abundance across all exposure stages and elevation zones, confirming its regional dominance (Table 1). Differences between T1 and T2 and between T1 and T3 were mainly driven by *Daucus carota* and *Astragalus sinicus* , whereas differences between T2 and T3 were primarily associated with *C. thunbergii* and *Daucus carota* . Spatial differences between HE and both ME and LE were largely driven by *Artemisia selengensis* , whereas dissimilarity between ME and LE was mainly attributed to *Daucus carota* , *Ranunculus polii*, and *Astragalus sinicus* (Table 2).

**TABLE 1:** Key driver species and their contributions to plant community dissimilarity across exposure stages and elevation zones.

Species	Average abundance/contribution (%)					
	T1	T2	T3	HE	ME	LE
*Ranunculus polii*	0.63/2.95	1.72/12.04	3.01/3.54	—	1.76/6.29	3.74/18.80
*Carex thunbergii*	18.23/86.50	14.08/44.69	28.66/40.61	28.13/59.62	18.88/56.96	13.96/60.92
*Astragalus sinicus*	—	1.74/11.09	5.74/14.21	1.64/3.65	3.71/10.84	—
*Daucus carota*	—	22.80/17.08	7.11/16.57	3.69/8.21	3.39/9.44	2.83/5.61
*Artemisia selengensis*	—	—	—	8.35/8.51	—	—
*Medicago sativa*	0.4/2.83	—	—	—	—	—
*Poa annua*	—	0.92/4.03	1.69/3.55	—	—	1.30/5.78
*Galium bungei*	—	2.34/3.24	1.81/2.91	1.41/3.89	1.34/3.82	—
*Leonurus japonicus*	—	—	1.87/2.68	—	—	—

**TABLE 2:** Species‐specific contributions to differences among assemblage groups (bold values indicate contributions > 10%).

Species	Average dissimilarity/contribution (%)					
	T1&T2	T1&T3	T2&T3	HE&ME	HE&LE	ME&LE
*Ranunculus polii*	**6.16/10.57**	5.62/8.43	4.55/8.75	3.59/6.45	**6.20/10.12**	**6.34/12.26**
*Potentilla supina*	3.28/5.62	3.23/4.84	3.16/6.08	3.38/6.07	1.86/3.04	4.40/8.50
*Astragalus sinicus*	**6.55/11.23**	**8.26/12.39**	4.63/8.90	5.07/9.10	4.29/7.00	**6.28/12.13**
*Daucus carota*	**8.63/14.79**	**9.27/13.91**	4.79/9.21	5.30/9.52	5.88/9.60	**6.44/12.45**
*Galium bungei*	3.33/5.71	3.46/5.19	3.35/6.44	3.60/6.48	3.26/5.32	3.29/6.36
*Galium hoffmeisteri*	—	3.23/4.85	2.83/5.45	2.30/4.12	1.75/2.85	1.26/2.44
*Lapsanastrum apogonoides*	—	2.39/3.58	2.09/4.02	1.12/2.02	0.97/1.59	1.63/3.15
*Leonurus japonicus*	3.23/5.54	3.30/4.96	3.13/6.02	3.64/6.54	4.31/7.04	2.58/4.99
*Carex thunbergii*	**5.91/10.13**	4.04/6.06	4.82/9.27	4.07/7.32	5.85/9.54	4.90/9.47
*Poa annua*	4.00/6.86	3.75/5.63	3.39/6.51	2.48/4.46	3.71/6.05	4.14/7.99
*Artemisia selengensis*	5.01/8.60	4.20/6.30	2.43/4.66	**6.45/11.59**	**7.13/11.64**	—
*Persicaria criopolitana*	2.73/4.68	2.10/3.15	1.09/2.10	1.04/1.88	2.55/4.16	2.67/5.16
*Persicaria lapathifolia*	—	4.17/6.26	3.60/6.92	1.81/3.26	2.06/3.36	2.10/4.06
*Youngia japonica*	2.13/3.65	—	1.35/2.60	1.61/2.89	1.53/2.49	—
*Medicago sativa*	2.07/3.54	1.40/2.09	—	1.46/2.62	—	1.73/3.34
*Calystegia hederacea*	—	1.52/2.28	0.99/1.90	1.68/3.02	1.88/3.07	—
*Trigonotis peduncularis*	—	0.94/1.41	—	—	—	—
*Rumex dentatus*	—	—	1.43/2.75	1.85/3.32	2.05/3.35	—

### 3.3 Relationship between Species Diversity and Soil Factors

**FIGURE 6:**
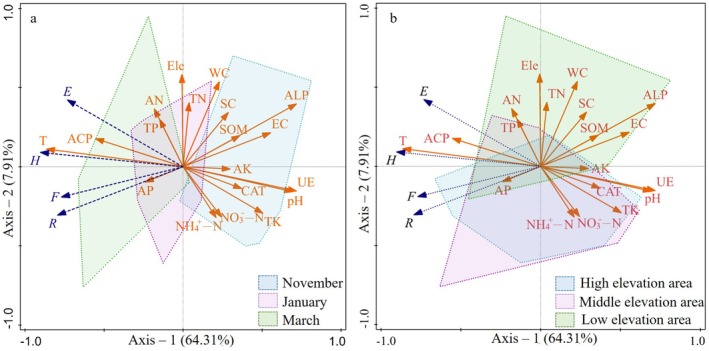
Influence of environmental factors on plant community diversity based on RDA. The figure illustrates RDA results across temporal (a) and spatial (b) dimensions. Panel (a) shows the temporal ordination for November, January, and March, with polygons enclosing samples from each month. Panel (b) presents the spatial ordination for high‐, middle‐, and low‐elevation zones, with polygons representing distinct elevation gradients. Environmental variables include electrical conductivity (EC), water content (WC), pH, soil organic matter (SOM), total nitrogen (TN), available nitrogen (AN), ammonium nitrogen (NH_4_^+^‐N), nitrate nitrogen (NO_3_^−^‐N), total phosphorus (TP), alkaline phosphatase (AP), total potassium (TK), available potassium (AK), catalase (CAT), alkaline phosphatase (ALP), acid phosphatase (ACP), urease (UE), sucrase (SC), elevation gradient (Ele) and exposure duration (T). The RDA analysis highlights key interactions between plant communities and their environment across both temporal and spatial dimensions.

To examine the effects of soil factors on plant community diversity, RDA was performed with community diversity indices and species composition as response variables, and elevation, exposure duration, and soil variables as explanatory factors. The first ordination axis explained 64.31% of the variance in the relationship between diversity indices and environmental factors, whereas the second axis explained 7.91%. The cumulative explanatory power of the first two axes reached 72.22% (Figure 6). Exposure duration accounted for the largest proportion of variation in plant community diversity indices (65.40%, *p* < 0.01), followed by ALP (7.80%), NO_3_^−^‐N (7.60%), and pH (5.30%) (all *p* < 0.05) (Table S5 in the Data S1). The overall RDA model was significant (*F* = 3.9, *p* < 0.01) (Table 3), indicating a robust explanation of the relationship between plant community characteristics and soil factors. Exposure duration, ACP, AN, AP, and TP were positively correlated with diversity indices and species richness, whereas SC, SOM, ACP, EC, UE, pH, CAT, TK, NH_4_^+^‐N, and NO_3_^−^‐N were negatively correlated. Temporally, samples from March (T3) and January (T2) showed substantial overlap (Figure 6a). Spatially, samples from the middle–and HE also overlapped considerably (Figure 6b).

**TABLE 3:** RDA ordination of plant community diversity indices and soil properties in the Shengjin Lake intertidal wetland, China.

RDA ordination	Axis—1	Axis—2	Axis—3	Axis—4
Eigenvalues	0.6431	0.0791	0.0153	0.0011
Explained variation	64.31	72.22	73.74	73.85
Pseudo‐canonical correlation	0.8851	0.7236	0.7737	0.6439
Explained fitted variation	87.08	97.79	99.86	100.00
Test of significance of all canonical axes	*p* = 0.02			

**FIGURE 7:**
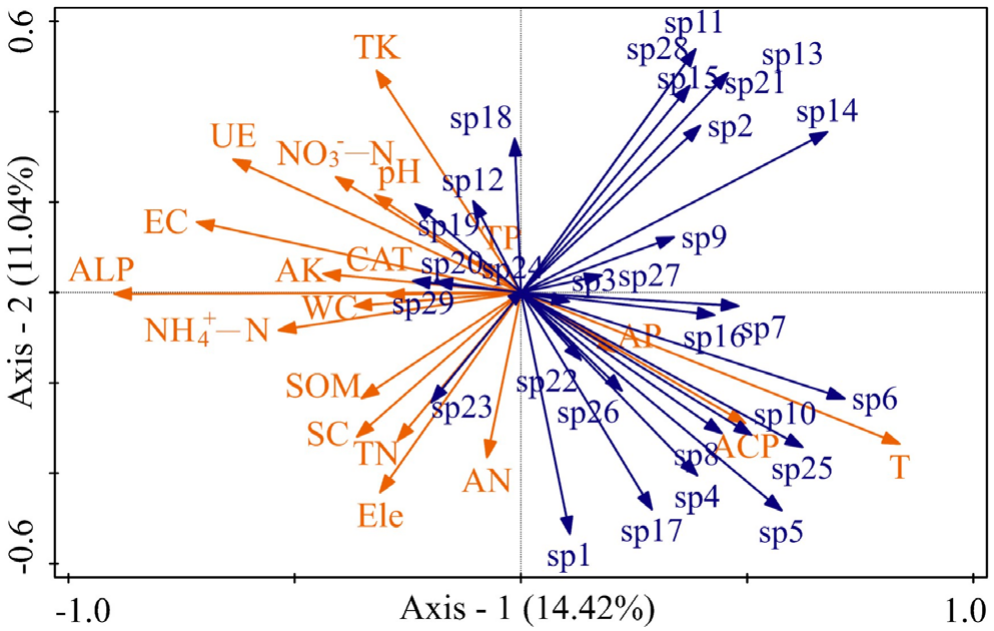
Effects of environmental factors on plant species distribution based on RDA. The figure shows the RDA ordination illustrating relationships between environmental variables and the distribution of 29 plant specie**s**. Environmental factors include electrical conductivity (EC), water content (WC), pH, soil organic matter (SOM), total nitrogen (TN), available nitrogen (AN), ammonium nitrogen (NH_4_^+^‐N), nitrate nitrogen (NO_3_^−^‐N), total phosphorus (TP), available phosphorus (AP), total potassium (TK), available potassium (AK), catalase (CAT), alkaline phosphatase (ALP), acid phosphatase (ACP), urease (UE), sucrase (SC), elevation gradient (Ele) and exposure duration (T). Species (sp) numbered 1–29 representing *Ranunculus polii*, *Rumex dentatus, Geranium carolinianum, Potentilla supina, Astragalus sinicus, Daucus carota, Galium bungei, Galium hoffmeisteri, Erigeron canadensis, Lapsanastrum apogonoides, Pseudognaphalium affine, Youngia japonica, Trigonotis peduncularis, Leonurus japonicus, Salvia plebeia, Carex thunbergii, Poa annua, Artemisia selengensis, Medicago sativa, Ranunculus sceleratus, Calystegia hederacea, Stellaria media, Persicaria criopolitana, Hemisteptia lyrata, Bothriospermum zeylanicum, Rorippa amphibia*, and *Persicaria hydropiper*.

RDA was further applied to evaluate relationships between plant species distribution and environmental factors. The first ordination axis explained 14.42% of the variance in species–environment relationships, and the second axis explained 11.04%, with a cumulative explanatory rate of 25.46% (Figure 7). Differences among environmental factors were highly significant (*F* = 1.6, *p* < 0.01) (Table 4). ALP exerted the strongest effect on species distribution, accounting for 22.40% of the variance (*p* < 0.01), followed by SOM (8.40%) and EC (5.60%) (both *p* < 0.01). Elevation and exposure duration also significantly affected species distribution (both *p* < 0.01), explaining 11.40% and 8.40% of the variance, respectively (Table S6 in the Data S1).

**TABLE 4:** RDA ordination of plant species and soil properties in the Shengjin Lake intertidal wetland, China.

RDA ordination	Axis—1	Axis—2	Axis—3	Axis—4
Eigenvalues	0.1442	0.1104	0.0623	0.0362
Explained variation	14.42	25.46	31.69	35.41
Pseudo‐canonical correlation	0.8999	0.8436	0.8506	0.8039
Explained fitted variation	26.98	47.64	59.30	66.07
Test of significance of all canonical axes	*p* = 0.02			

## 4 Discussion

### 4.1 Spatiotemporal Dynamics of Plant Communities Along the Elevation Gradient During the Exposure Period

During the exposure period, flooding stress associated with the elevation gradient represents the primary driver of spatial differentiation in plant communities in the Shengjin Lake intertidal wetland. The hydrological gradient filters species by tolerance and life‐history strategy, thereby shaping community composition and structure. In terms of species composition, the dominance of *C. thunbergii* reflects its high tolerance to inundation stress, which enables it to persist across both frequently flooded LE and long‐exposed HE. *C. thunbergii* is a typical clonal species with a well‐developed root system (Yuan et al. 2019), conferring strong anchorage and resistance to hydraulic scouring. Previous studies have shown that this species can survive flooding stress for up to 6 months (Hu et al. 2024), indicating substantial submergence tolerance. With increasing elevation, flooding disturbance weakens and exposure duration increases, leading to a higher proportion of perennial species, such as *A. selengensis* . This shift corresponds to more stable moisture conditions in HE, which favor biomass accumulation and population persistence of perennial plants. By contrast, annual species in LE, such as *Poa annua* , exhibit rapid life cycles that allow completion of reproduction before inundation (Stromberg et al. 2017), thereby supporting population persistence under frequent flooding.

The spatiotemporal variation in plant community diversity further reflects hydrological gradient–plant interactions under different exposure durations. As an integrated environmental factor, elevation drives concurrent changes in light availability, water regime, and soil conditions over relatively short distances, thereby affecting plant performance and community diversity (Sim‐Sim et al. 2015; Sleeper and Ficklin 2016; Zhang et al. 2022). Previous studies have reported that increasing inundation depth decreases plant biomass (Huang et al. 2021; Xi et al. 2025). During the early exposure stage, the LE showed the lowest species richness, likely due to frequent inundation (Chen et al. 2024). Additionally, hypoxia and substrate scouring associated with prolonged flooding can inhibit seed bank germination (Zhou et al. 2020). During the middle exposure stage, *H*, *F*, and *E* were lower in the HE than in the ME. This pattern can be partly attributed to the high coverage of *C. thunbergii* , which imposes strong light limitation on subordinate species (Ou et al. 2025). In relatively stable environments with sufficient light resources, dominant species can further restrict coexistence by rapidly occupying available niches (Burkle and Belote 2015; Ren et al. 2024). By the late exposure stage, diversity indices and species richness peaked in the ME (Figure 4). As a transition zone between frequently flooded and rarely flooded areas, the ME experiences intermediate hydrological disturbance. Such moderate disturbance disrupts single‐species dominance and promotes species coexistence (Guo et al. 2019). In the HE, greater height and density of *C. thunbergii* and *A. selengensis* increase competitive exclusion. Higher plant density is generally associated with lower species diversity (Han et al. 2020), explaining the lower *H* values in the HE compared with the ME.

### 4.2 Spatiotemporal Variations of Soil Properties and Enzyme Activities Along the Elevation Gradient During the Exposure Period

Distinct exposure stages in the intertidal wetland drove significant spatiotemporal variation in soil properties along the elevation gradient. In the HE, EC initially increased during early exposure as evaporative water loss led to relative enrichment of base cations. EC then declined as precipitation‐driven leaching exceeded evaporation, combined with plant uptake and soil adsorption processes (Omogbehin and Oluwatimilehin 2022; Sui et al. 2025). In the ME and LE, EC decreased continuously because of sustained leaching. Although WC generally declined owing to increasing evapotranspiration and drainage, the LE maintained relatively high moisture levels because delayed water recession continuously replenished surface soils (Bao et al. 2025). The biogeochemical cycling of nitrogen, phosphorus, and potassium in wetland soils is strongly regulated by exposure duration and elevation (Li et al. 2017; Sola et al. 2018; Gao et al. 2020). During early exposure, improved soil aeration in LE enhanced organic nitrogen mineralization and nitrification, generating transient increases in NH_4_^+^‐N and NO_3_^−^‐N, consistent with substrate‐limited dynamics reported by Mayela et al. (2021). In the late exposure stage, reduced soil moisture suppressed microbial activity, whereas enhanced plant uptake and intensified leaching further reduced NH_4_^+^‐N and NO_3_^−^‐N concentrations (Sardans et al. 2008; Długosz et al. 2023). Similar processes operated in the ME and HE. TN increased progressively due to cumulative inputs from plant residues (Fang‐di et al. 2023), whereas AN was continuously replenished by soluble organic nitrogen released during mineralization. TP exhibited a unimodal pattern, increasing during early exposure as aerobic conditions promoted organic phosphorus mineralization, followed by a decline after depletion of labile organic phosphorus pools (Xi et al. 2020). Concurrently, ALP produced by microorganisms enhanced organic phosphorus hydrolysis, temporarily increasing TP under aerated rhizosphere conditions (Xi et al. 2020; Li et al. 2024). AP remained relatively stable because of soil buffering through adsorption–desorption equilibrium.

Soil enzymes regulate wetland biogeochemical processes and respond sensitively to environmental conditions (Wang et al. 2019). ALP activity decreased with increasing exposure duration, reflecting reduced synthesis associated with metabolic shifts in aerobic microorganisms following water recession. This decline indicates a reduced potential for organic phosphorus mineralization, consistent with the decreasing TP observed at later stages. Increasing ACP activity, concurrent with declining pH (Balbaied and Moore 2019), suggests a compensatory role in sustaining phosphorus mineralization under more acidic conditions. UE activity decreased continuously, indicating reduced urea hydrolysis and nitrogen supply, which corresponds to decreases in NH_4_^+^‐N and reflects coordinated attenuation of nitrogen transformation. Elevated SC activity in the ME and LE was associated with greater inputs of root exudates as carbon substrates and enhanced microbial activity, which accelerated organic matter decomposition and supported nutrient conversion. By contrast, CAT activity showed a limited response to short‐term drying–rewetting cycles and remained relatively stable.

The LE experienced stronger redox fluctuations and material transport, including leaching and sediment deposition, because of frequent inundation. These processes resulted in greater variability in nutrient concentrations and enzyme activities. In contrast, the ME and HE experienced longer exposure durations and more stable moisture conditions, and soil processes were dominated by biological regulation, such as microbial metabolism, resulting in smoother temporal changes. This contrast illustrates how exposure duration shapes microenvironmental heterogeneity along elevation gradients and ultimately governs the spatiotemporal differentiation of soil properties and enzyme activities.

### 4.3 Interaction Mechanisms Within the Plant–Soil–Enzyme System in Response to Elevation Gradients and Exposure Period Dynamics

Plant community composition and structure directly reflect habitat characteristics jointly regulated by intertidal exposure duration and elevation gradients. The responses of species diversity to environmental drivers show significant spatiotemporal heterogeneity (Ngawang et al. 2025), highlighting the role of biodiversity in sustaining ecosystem function and services (Hector and Bagchi 2007). In intertidal wetlands, exposure duration represents the primary driver of plant community dynamics. The temporal sequence from T1 to T3 captures hydrological rhythms and serves as the main axis along which community responses occur. Differences in exposure duration modify the balance between facilitation and competition among plant species, consistent with the stress‐gradient hypothesis (SGH) (Daleo and Iribarne 2009; Luo et al. 2010). In agreement with the SGH, the present results indicate that longer exposure durations shift species interactions toward facilitation, thereby increasing species diversity and evenness.

In addition to temporal effects, elevation gradients from HE to LE generate spatial heterogeneity that shapes plant community structure. The effect of elevation depends on the position of the community along the stress gradient (Yang et al. 2015). As reported by Scrosati and Heaven (2007), species richness and diversity increase as environmental stress decreases, which is consistent with the observed patterns in this study. The soil environment, including physicochemical properties and enzyme activities, acts as a key mediator by translating hydrological signals into resource availability and stress conditions perceived by plants (Geng et al. 2017).

Among soil nutrients, NO_3_^−^‐N may reduce species diversity through multiple mechanisms. Elevated NO_3_^−^‐N can promote the competitive expansion of biennial herbs such as *Daucus carota* , which increased in abundance during the mid‐to‐late exposure stages (contribution > 15%, Table 1), thereby excluding species with narrower ecological niches (Green and Galatowitsch 2002). In LE, high NO_3_^−^‐N can act together with elevated EC to impose combined osmotic and ionic stress, further filtering out stress‐intolerant species. Moreover, denitrification under fluctuating exposure conditions can increase NO_3_^−^‐N variability, reducing nitrogen stability and weakening conditions for stable species coexistence (Zhang et al. 2023). Soil pH was negatively correlated with species diversity and richness. Variations in pH influence mineralization processes that regulate the conversion of organic matter into plant‐available nutrients, thereby indirectly shaping soil nutrient dynamics (Khan et al. 2019) and affecting plant growth and community diversity (Wang et al. 2014; Fan et al. 2019).

Soil enzyme activities affect plant community structure indirectly by controlling nutrient transformation processes (Xiao et al. 2021). Among the enzymes measured, ALP is a key indicator of phosphorus mineralization potential and availability (Li et al. 2021), and was the most influential enzyme affecting plant community patterns in this study (*p* < 0.01). In LE, hypoxic conditions promote microbial secretion of ALP to mineralize organic phosphorus, thereby satisfying microbial demand and increasing phosphorus availability for plants (Pang et al. 2024). This process supports the dominance of species such as *C. thunbergii* , whose root exudates may further stimulate microbial ALP production. SC catalyzes the decomposition of SOM, driving carbon cycling and enhancing soil fertility (Kotroczo et al. 2014). In the present study, SC activity increased gradually in the ME and LE with prolonged exposure, which may reflect increasing substrate availability. Root exudates released during plant growth supply carbon and nitrogen sources to microorganisms, promoting microbial activity and, in turn, accelerating organic matter decomposition (Brzostek et al. 2013). UE participates in the transformation of organic nitrogen into inorganic forms, and its activity declined progressively across all elevation zones with increasing exposure duration. During early exposure, microbial communities were less constrained, allowing UE to maintain moderate activity. As exposure continued, cumulative moisture loss imposed stress on microbial communities, leading to reduced UE activity and weakened nitrogen transformation. ACP showed relatively limited effects on plant community diversity in this study, suggesting a secondary role compared with other enzymes under the observed exposure conditions.

## 5 Conclusions

This study shows that prolonged exposure duration in the Shengjin Lake intertidal wetland significantly increases plant community species diversity. This pattern is primarily driven by exposure duration and further regulated by concurrent changes in soil conditions. Soil physicochemical properties and enzyme activities exhibited clear temporal and spatial variation, reflecting microbial‐mediated nutrient transformation processes that adjust to hydrological dynamics. These processes are tightly coupled. Extended exposure directly modifies soil moisture and redox conditions, thereby regulating microbial activity and function and influencing the efficiency and bioavailability of soil nutrient transformations. In turn, changes in nutrient availability filter plant species and shape community composition and diversity. Overall, the plant–soil–enzyme system exhibits a cascading pathway, from hydrological forcing (exposure duration and elevation gradient) to soil environmental responses (physicochemical properties and enzyme activities), ultimately shaping plant community succession.

## Author Contributions

**Jiaxin Li:** conceptualization (equal), data curation (equal), formal analysis (equal), investigation (equal), methodology (equal), software (lead), validation (lead), visualization (lead), writing – original draft (lead). **Wenwen Chen:** investigation (equal), methodology (equal), supervision (equal), validation (equal). **Zulan Ou:** investigation (equal), methodology (equal), supervision (equal), validation (equal). **Xingxing Yang:** investigation (equal), methodology (equal), supervision (equal). **Lingling Li:** formal analysis (equal), investigation (equal), methodology (equal), supervision (equal), validation (equal). **Chunlin Li:** funding acquisition (supporting), methodology (equal), writing – review and editing (equal). **Yansong Chen:** funding acquisition (lead), investigation (equal), methodology (equal), project administration (equal), resources (lead), supervision (lead), validation (equal).

## Funding

This work was supported by the Anhui Provincial Ecological Environment Monitoring Center (2022BFAFN02495), National Natural Science Foundation of China (32571751), the Young Elite Scientists Sponsorship Program of Anhui Association for Science and Technology (RCTJ202405), and the University Natural Science Research Project of Anhui Province (2024AH050074).

## Conflicts of Interest

The authors declare no conflicts of interest.

## Supporting information


**Data S1:** Supporting Information.

## Data Availability

All the required data are uploaded as Supporting Information.
