# Supplementary material for: Spatiotemporal Dynamics of Plant–Soil–Enzyme Interactions in Intertidal Wetlands
Source: Ecol Evol. 2026 Mar 14;16(3):e73259. doi: 10.1002/ece3.73259 (PMC13093767; doi:10.1002/ece3.73259)
Supplement: Supplementary file 1 — Data S1: Supporting Information. [file ECE3-16-e73259-s001.docx]

**Spatiotemporal dynamics of plant–soil–enzyme interactions in intertidal wetlands** for Ecology and Evolution

Jiaxin Li, Wenwen Chen, Zulan Ou, Xingxing Yang, Lingling Li, Yansong Chen

Corresponding author: Yansong Chen

Affiliation: School of Biology and Food Engineering, Hefei Normal University, Hefei 230061, China

E-mail address: [YSChen@hfnu.edu.cn](mailto:YSChen@hfnu.edu.cn)

Table S1: Spatiotemporal dynamics of soil physicochemical properties across exposure stages and elevation gradients in Shengjin Lake intertidal wetland.

| Index | Elevation | | Exposure duration | | | Elevation 🞩 Exposure duration | |
| --- | --- | --- | --- | --- | --- | --- | --- |
|  | *F* | *P* | *F* | | *P* | *F* | *P* |
| EC | 2.373 | 0.135 | 55.296 | <0.001 | | 3.106 | 0.048 |
| WC | 2.534 | 0.121 | 8.203 | 0.006 | | 0.824 | 0.495 |
| pH | 0.455 | 0.645 | 26.282 | <0.001 | | 0.019 | 0.991 |
| SOM | 1.027 | 0.388 | 2.965 | 0.091 | | 0.407 | 0.742 |
| TN | 2.671 | 0.11 | 0.960 | 0.383 | | 1.866 | 0.165 |
| AN | 2.568 | 0.118 | 19.005 | <0.001 | | 2.936 | 0.047 |
| NH_4_^+^-N | 3.394 | 0.068 | 11.970 | <0.001 | | 1.712 | 0.18 |
| NO_3_^-^-N | 1.697 | 0.224 | 9.478 | 0.005 | | 5.295 | 0.013 |
| TP | 0.046 | 0.955 | 16.726 | <0.001 | | 0.727 | 0.552 |
| AP | 2.384 | 0.134 | 0.636 | 0.528 | | 0.147 | 0.956 |
| TK | 0.491 | 0.624 | 9.259 | 0.002 | | 1.832 | 0.165 |
| AK | 0.67 | 0.53 | 3.516 | 0.056 | | 1.211 | 0.334 |

Table S2: Spatiotemporal dynamics of soil enzyme activity across exposure stages and elevation gradients in Shengjin Lake intertidal wetland.

| Index | Elevation | | Exposure duration | | | Elevation 🞩 Exposure duration | |
| --- | --- | --- | --- | --- | --- | --- | --- |
|  | *F* | *P* | *F* | | *P* | *F* | *P* |
| CAT | 0.663 | 0.533 | 4.332 | 0.059 | | 1.058 | 0.377 |
| ALP | 2.566 | 0.118 | 48.327 | <0.001 | | 1.802 | 0.179 |
| ACP | 1.923 | 0.118 | 20.871 | <0.001 | | 0.314 | 0.795 |
| SC | 5.891 | 0.017 | 0.18 | 0.802 | | 0.115 | 0.963 |
| UE | 1.849 | 0.2 | 58.02 | <0.001 | | 0.748 | 0.517 |

Table S3: Species composition in Shengjin Lake intertidal wetland during the exposure period.

| Serial number | Latin name | Family | Genera | Life-style |
| --- | --- | --- | --- | --- |
| 1 | *Erigeron canadensis* | Asteraceae | *Erigeron* | A |
| 2 | *Youngia japonica* | Asteraceae | *Youngia* | A |
| 3 | *Lapsanastrum apogonoides* | Asteraceae | *Lapsanastrum* | A |
| 4 | *Hemisteptia lyrata* | Asteraceae | *Hemisteptia* | A |
| 5 | *Pseudognaphalium affine* | Asteraceae | *Pseudognaphalium* | A |
| 6 | *Taraxacum mongolicum* | Asteraceae | *Taraxacum* | P |
| 7 | *Artemisia selengensis* | Asteraceae | *Artemisia* | P |
| 8 | *Rumex dentatus* | Brassicaceae | *Rorippa* | A |
| 9 | *Persicaria lapathifolia* | Polygonaceae | *Persicaria* | A |
| 10 | *Persicaria hydropiper* | Polygonaceae | *Persicaria* | A |
| 11 | *Persicaria criopolitana* | Polygonaceae | *Persicaria* | A |
| 12 | *Ranunculus sceleratus* | Ranunculaceae | *Ranunculus* | A |
| 13 | *Ranunculus polii* | Ranunculaceae | *Ranunculus* | A |
| 14 | *Salvia plebeia* | Lamiaceae | *Salvia* | A/B |
| 15 | *Leonurus japonicus* | Lamiaceae | *Leonurus* | A/B |
| 16 | *Galium hoffmeisteri* | Rubiaceae | *Galium* | A |
| 17 | *Galium bungei* | Rubiaceae | *Galium* | P |
| 18 | *Astragalus sinicus* | Fabaceae | *Astragalus* | B |
| 19 | *Medicago sativa* | Fabaceae | *Medicago* | P |
| 20 | *Trigonotis peduncularis* | Boraginaceae | *Trigonotis* | B |
| 21 | *Bothriospermum zeylanicum* | Boraginaceae | *Bothriospermum* | A |
| 22 | *Stellaria media* | Caryophyllaceae | *Stellaria* | A/B |
| 23 | *Poa annua* | Gramineae | *Poa* | A |
| 24 | *Potentilla supina* | Rosaceae | *Potentilla* | A/B |
| 25 | *Daucus carota* | Apiaceae | *Daucus* | B |
| 26 | *Carex thunbergii* | Cyperaceae | *Carex* | P |
| 27 | *Calystegia hederacea* | Convolvulaceae | *Calystegia* | A |
| 28 | *Geranium carolinianum* | Geraniaceae | *Geranium* | A |
| 29 | *Rorippa amphibia* | Brassicaceae | *Rorippa* | P |

Note: A, B and P represent Annual herb, Biennial plant and Perennial herb, respectively.

Table S4: Spatiotemporal dynamics of species diversity and richness of plant community across exposure stages and elevation gradients in Shengjin Lake intertidal wetland.

| Index | Elevation | | Exposure duration | | | Elevation 🞩 Exposure duration | |
| --- | --- | --- | --- | --- | --- | --- | --- |
|  | *F* | *P* | *F* | | *P* | *F* | *P* |
| *F* | 3.878 | 0.033 | 53.903 | <0.001 | | 1.455 | 0.233 |
| *H* | 1.584 | 0.224 | 174.663 | <0.001 | | 3.541 | 0.015 |
| *E* | 1.050 | 0.364 | 83.631 | <0.001 | | 3.812 | 0.035 |
| *R* | 8.692 | 0.001 | 92.024 | <0.001 | | 1.537 | 0.207 |

Table S5: RDA ordination reveals the effects of environmental factors on plant diversity in the Shengjin Lake intertidal wetland

| Name | Explains（ %） | Contribution（ %） | Pseudo-*F* | *P* |
| --- | --- | --- | --- | --- |
| T | 48.3 | 65.4 | 40.2 | 0.002 |
| ALP | 5.8 | 7.8 | 5.3 | 0.008 |
| NO_3_^-^-N | 5.6 | 7.6 | 5.7 | 0.012 |
| pH | 3.9 | 5.3 | 4.3 | 0.02 |
| NH_4_^+^-N | 2.0 | 2.7 | 2.3 | 0.106 |
| Ele | 1.6 | 2.1 | 1.8 | 0.174 |
| SC | 1.5 | 2.0 | 1.8 | 0.192 |
| TP | 1.2 | 1.6 | 1.4 | 0.236 |
| WC | 0.7 | 1.0 | 0.9 | 0.374 |
| EC | 0.9 | 1.2 | 1.1 | 0.348 |
| AK | 0.5 | 0.7 | 0.6 | 0.508 |
| ACP | 0.4 | 0.6 | 0.5 | 0.62 |
| UE | 0.3 | 0.4 | 0.3 | 0.772 |
| AP | 0.2 | 0.3 | 0.3 | 0.762 |
| TK | 0.3 | 0.5 | 0.4 | 0.67 |
| AN | 0.3 | 0.4 | 0.3 | 0.732 |
| CAT | < 0.1 | 0.1 | < 0.1 | 0.936 |
| TN | < 0.1 | < 0.1 | < 0.1 | 0.974 |
| SOM | < 0.1 | < 0.1 | < 0.1 | 0.958 |

Table S6: RDA ordination reveals the effects of environmental factors on species importance value in the Shengjin Lake intertidal wetland

| Name | Explains（ %） | Contribution（ %） | Pseudo-*F* | *P* |
| --- | --- | --- | --- | --- |
| ALP | 12.0 | 22.4 | 5.9 | 0.002 |
| Ele | 6.1 | 11.4 | 3.1 | 0.002 |
| T | 4.5 | 8.4 | 2.4 | 0.002 |
| SOM | 3.7 | 6.9 | 2.0 | 0.06 |
| EC | 3.0 | 5.6 | 1.7 | 0.072 |
| pH | 2.6 | 4.9 | 1.5 | 0.178 |
| NH_4_^+^-N | 2.9 | 5.4 | 1.6 | 0.078 |
| TP | 2.3 | 4.3 | 1.3 | 0.178 |
| TK | 1.9 | 3.5 | 1.1 | 0.348 |
| TN | 2.2 | 4.1 | 1.3 | 0.222 |
| WC | 1.7 | 3.2 | 1.0 | 0.442 |
| AN | 1.6 | 3.1 | 1.0 | 0.43 |
| NO_3_^-^-N | 1.3 | 2.5 | 0.8 | 0.714 |
| AP | 1.5 | 2.9 | 0.9 | 0.578 |
| AK | 1.4 | 2.6 | 0.8 | 0.664 |
| UE | 1.2 | 2.2 | 0.7 | 0.724 |
| SC | 1.2 | 2.2 | 0.6 | 0.832 |
| ACP | 1.1 | 2.1 | 0.6 | 0.824 |
| CAT | 1.2 | 2.2 | 0.6 | 0.818 |
